# RNAi‐mediated gene silencing of a 26S proteasome subunit increases mortality of the Japanese beetle *Popillia japonica*

**DOI:** 10.1002/ps.70265

**Published:** 2025-10-06

**Authors:** Giulia Lucetti, Simona Abbà, Gabriele Pesavento, Elena Fanelli, Francesca De Luca, Elia Battagini, Stefano Cazzaniga, Matteo Ballottari, Diletta Frizzon, Nicola Mori, Luciana Galetto

**Affiliations:** ^1^ Institute for Sustainable Plant Protection ‐ IPSP National Research Council – CNR Torino Italy; ^2^ Department of Biotechnology University of Verona Verona Italy; ^3^ Institute for Sustainable Plant Protection ‐ IPSP National Research Council – CNR Bari Italy

**Keywords:** pest control, RNA interference (RNAi), double‐stranded RNA (dsRNA), dsRNA delivery, regulatory particle non‐ATPase 6, shibire_dynamin‐like protein

## Abstract

**BACKGROUND:**

The Japanese beetle *Popillia japonica* (Coleoptera: Scarabaeidae) is a highly polyphagous quarantine invasive species causing severe crop damages. Its management is based on broad‐spectrum insecticides and sustainable alternatives are needed. Strategies based on RNA interference (RNAi) emerged in crop protection and we aimed to explore its use to control *P. japonica*.

**RESULTS:**

Nine genes of *P. japonica* were selected as lethal candidates, based on previous wide‐genome screenings on other coleopterans. To avoid off‐target effects, genes showing over 80% identity with pollinator homologues were excluded and *P. japonica* double‐stranded RNAs (dsRNAs) were designed in the least conserved portions according to alignments with *Apis mellifera*. When incubated in *P. japonica* midgut juice, dsRNAs were not degraded. Injection and plant‐mediated feeding were used to deliver dsRNAs to larvae and adults. Five targets were tested, and two genes were selected as the most effective in increasing mortality, namely regulatory particle non‐ATPase 6 subunit (RPN) and shibire_dynamin‐like protein (SHI). A significant transcript reduction up to 21 days (RPN: 3–5 fold‐change silencing) after dsRNA injection indicated that effective gene silencing occurred, as also supported by sequencing of small RNA libraries. In adults, RNAi‐mediated depletion of RPN transcript reduced survival, either when insects were injected or mass‐fed on vine leaves dsRNA‐treated.

**CONCLUSION:**

A subunit of the 26S proteasome was indicated as promising RNAi target for dsRNA‐based insecticide against the Japanese beetle. The data pave the way for the possible use of RNAi approaches to control this pest, proactively waiting for the European Union approval of exogenously applied dsRNAs. © 2025 The Author(s). *Pest Management Science* published by John Wiley & Sons Ltd on behalf of Society of Chemical Industry.

## Graphical abstract

We propose the regulatory particle non‐ATPase subunit 6 of the 26S proteasome as a first good promising RNA interference target candidate to control the Japanese beetle *Popillia japonica*. This will help to optimize a double‐stranded RNA insecticide against this priority pest with low off‐target effects on pollinators on a gene sequence‐based prediction.

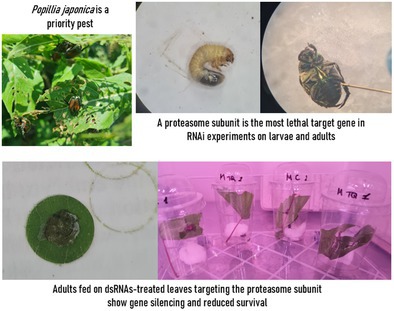

## 1 INTRODUCTION

The Japanese beetle, *Popillia japonica* Newman (Coleoptera: Scarabaeidae) is an invasive pest of agricultural crops, ornamental plants, lawns and forests.^1^ Being able to feed on more than 400 host plant species, it is one of the most polyphagous plant pests.^2^ *Popillia japonica* is a quarantine species, listed in Annex I Part A/1 of Council Directive 2000/29/EC, included in the European Plant Protection Organization (EPPO) list A2,^2^, ^3^ and indexed among the most crucial potential priority pest in Europe.^4^, ^5^ Originally from Japan, in the early 1900s *P. japonica* spread in the United States and Canada, in the 1970s in the Azores and in the 21st century in Europe. Interestingly, the mitochondrial genetic variability of different *P. japonica* worldwide populations revealed that the invasive samples of the United States derived from multiple introduced Japanese lineages^6^ and that the Azorean and European populations are independent introductions from North America.^7^ Two *P. japonica* outbreaks are known to have occurred in the old continent: Lombardy region (Italy) in 2014, and Ticino canton (Switzerland) in 2017.^8^ These European outbreaks continue to expand, and the pest is now established in other northern Italian regions (Piedmont, Aosta Valley, Emilia Romagna and Friuli Venezia Giulia)^9^ and in two other Swiss cantons (Valais and Zürich).^10^ *Popillia japonica* adults are active fliers and hitchhikers, and the spread of this pest is indeed a mix of natural dispersal and human‐assisted movements.^11^ Based on abundance data of *P. japonica* adults in Lombardy (Italy) from 2015 to 2021, the mean rate of expansion resulted to be 8.2 km per year, with fluctuations along different directions.^12^

*Popillia japonica* is generally univoltine with three larval instars. The beetle spends much of its life underground as eggs, three larval stages and pupae, and only a brief period above ground as adults during summer.^13^, ^14^ Eggs are laid in moist grassland in the summer. Larvae feed on roots throughout the autumn and spring, overwintering 15–20 cm belowground, and then pupate nearer the surface, emerging as adults.^14^ The potential damage costs due to *P. japonica* infestations can be considerable. In the United States the impact of the Japanese beetle on turf and ornamentals has been estimated to be US$ 450 million per year.^15^ In Europe heterogeneous effects across different countries and crops have been potentially predicted: for example, in major vine‐growing and wine‐producing countries, such as France and Italy, an eventual damage ranging from about EUR 65 to 90 million per year has been estimated.^16^, ^17^

Chemical, physical and biological measures have been, and are still being, used to suppress *P. japonica* populations.^4^, ^14^ Among them, application of broad‐spectrum chemical insecticides, Long‐Lasting Insecticide‐treated Nets, attract‐and‐kill systems, insect‐proof nets as well as experimental auto‐disseminating devices to spread entomopathogenic fungi (*Metarhizium* and *Beauveria* spp.) and nematodes (Heterorhabditidae and Steinernematidae) are noteworthy.^18^ Although not always effective, for the time being these are the main strategies used to limit *P. japonica* populations.^2^, ^4^ One of the major drawbacks is that applications of broad‐spectrum insecticides need to be repeated many times during the adult flight period, with obvious disadvantages for beneficial arthropod species and human health. Moreover, organic insecticide compounds were nearly not effective under any tested condition.^4^

Taken together the spread ability of *P. japonica*, its potential impact, the limited effectiveness of biological agents and the current limitation of chemical products registered against this pest, innovative and more effective, selective, sustainable and environmental‐friendly strategies are urgently needed to cope with the Japanese beetle. Pest control strategies based on RNA interference (RNAi) meet all these required criteria^19^ and evidence for its occurrence in *P. japonica* have been gathered.^20^ Briefly, RNAi is a post‐transcriptional gene silencing mechanism that can be triggered by delivering double‐stranded RNA (dsRNA) molecules to a target organism. The exogenous application of dsRNAs can silence an essential gene of the target pest and can be exploited as an innovative insecticide approach.^21^ The efficacy of the RNAi response induced by dsRNA treatment varies considerably among insect orders, with Coleoptera being the most sensitive and Diptera and Lepidoptera being the least responsive.^22^ The recent approval of the first sprayable dsRNA‐based insecticide product against the Colorado potato beetle *Leptinotarsa decemlineata* (Coleoptera: Chrysomelidae) demonstrates the promise of this approach and its effectiveness against beetles.^23^, ^24^ Nevertheless, the selection of pest genes to be silenced is a crucial step in the workflow for the development of an RNAi‐based insecticide strategy.^25^ Unbiased screenings surely are superior methods for discovering the best target processes and genes. This approach indeed excludes issues related to abundance and turn‐over of silenced transcript and cognate protein, redundancy of target mechanism by alternative pathways, or compensatory up‐regulation.^26^ Based on a set of genome‐wide screenings of the red flour beetle *Tribolium castaneum* (Coleoptera: Tenebrionidae),^27^, ^28^, ^29^ the most effective RNAi genes turned out to be those related to basic cellular functions, such as translation and proteasome‐mediated protein degradation, rather than those of nervous system, which conversely are the most popular targets of conventional chemical insecticides.^26^ Another advantage of RNAi‐based control strategies is the specificity of the sequence underlying the mechanism, which allows the risk to beneficial organisms to be avoided already from the dsRNA design phase.^30^ In addition, the genome of *P. japonica* was recently re‐sequenced and re‐assembled^31^ providing fundamental bioinformatic tools to drive such biotechnologically‐based control measures against this pest.

All these aspects prompted us to explore the possibility of using RNAi‐based strategies for the control of *P. japonica*. This work aims at (i) selecting the most promising candidate genes as RNAi targets, (ii) designing and producing specific dsRNAs to avoid undesirable effects on pollinators, (iii) setting up laboratory‐scale suitable protocols to treat larvae and adults and evaluate survival, (iv) optimizing molecular tools to evaluate gene silencing, (v) obtaining information on small RNA (sRNA) profiles upon dsRNA treatment.

## 2 MATERIALS AND METHODS

### 2.1 Insect collection and maintenance under laboratory condition

Different developmental stages of *P. japonica* alive specimens were collected in four sampling campaigns organized from August 2023 to September 2024 in a natural infested area of northwest Italy (Table 1). Alive adults and larvae were collected from pheromone traps and soil, respectively, and rapidly submersed with RNAlater (Sigma‐Aldrich, St Louis, MO, USA) in 15 mL plastic vials to preserve integrity of single strand nucleic acids. Specimens were stored at 4 °C before being dried with a clean towel, separately transferred into fresh tubes, flash‐freezed in liquid nitrogen and then stored at −80 °C until RNA extraction. Insects collected in 2024 were used for RNAi‐bioassays. Adults were collected with pheromone traps, caged in a large bugdorm on potted grapevines and maintained in a climatic chamber at 20–25 °C with a16 h:8 h light/dark photoperiod. Larvae were collected by inspection of portions of ground from lawn and maize fields, kept in a plastic bag with original soil at 4 °C before microinjection, which was performed within a few days from the field collection.

**Table 1:** Details of insect sampling campaigns

Sampling period	Sampling site	Life stage^†^	Sampling size	Experimental purpose
August 2023	Bodio Lomnago (VA), 45.783413 N, 8.758812 E	A, L1	8 adults, 3 larvae	Optimization of RNA extraction, synthesis of double‐stranded RNA (dsRNA), selection of housekeeping genes
September 2023	Cavaglià (BI), 45.403366 N, 8.104824 E	L2, L3	34 larvae	
March/April 2024	Occhieppo Inferiore (BI), 45.543899 N, 8.031176 E	L3	~ 400 post‐wintering larvae	dsRNA microinjection assay to select target genes (screening experiment)
	Salussola (BI), 45.470235 N, 8.161752 E			
	Rovasenda (VC),45.528067 N, 8.303482 E			
June/July 2024	Lamporo (VC), 45.219384 N, 8.098201 E	A	~ 500 adults	*Ex vivo* degradation assay, dsRNA microinjection and plant‐mediated feeding assays to silence selected target genes
September 2024	Salussola (BI), 45.447269 N, 8.134467 E	L2, L3	160 pre‐wintering larvae	dsRNA microinjection assays to silence selected target genes (over‐time silencing experiment) and sequencing of small RNAs

*Note*: Periods, sites, life stages, numbers of collected specimens and experimental purposes of *Popillia japonica* collection events.

^†^ A: adults; L1, L2, L3: larval stages.

### 2.2 Selection of target genes

Nine candidate *P. japonica* genes (Table 2) to be silenced by RNAi were chosen among those known to be potentially involved in lethal phenotype, according to previous wide‐genome studies on other coleopteran species.^27^, ^28^, ^29^ The selected genes were retrieved from the publicly available *P. japonica* genome assembly (BioProject: PRJNA860365) using *T. castaneum* as queries (Table 2, Supporting Information, Table S1). To exclude undesirable off‐target effects, coding sequences of the selected candidate *P. japonica* genes were aligned with homologues of *Apis mellifera*, and genes with identity higher than 80% with pollinator homologues were excluded from the subsequent design of dsRNAs.

**Table 2:** Selection of target genes

Gene name and abbreviation	*Tribolium castaneum* accession	*Popillia japonica* accession (protein)	Identity with *Apis mellifera* (CDS)	Size of *Popillia japonica* dsRNAs	Identity with *Apis mellifera* (dsRNAs)	n‐mer matches (nt)
Regulatory particle non‐ATPase 6 (RPN)	TC006375	KAK9686934.1	71%	388	67%	1 (10 nt), 1 (14 nt), 1 (15 nt)
Shibire_dynamin‐like protein (SHI)	TC011058	KAK9744413.1	76%	395	71%	1 (10 nt), 2 (11 nt), 1 (14 nt)
Coat protein (coatomer) β’ (COPI)	TC013867	KAK9737135.1	70%	384	74%	1 (10 nt), 2 (11 nt)
ras opposite (ROP)	TC011120	KAK9710256.1	73%	343	69%	1 (10 nt), 1 (11 nt), 1 (14 nt)
V‐type proton ATPase sub.d 1_(vATPaseD)	TC013357	KAK9746943.1	74%	367	74%	2 (11 nt), 1 (14 nt)
Signal recognition particle 54k (SRP54)	TC002574	KAK9711424.1	76%	348	76%	1 (10), 1 (11), 1 (14), 1 (21)
Tubulin beta‐1 chain‐like (betaTUB)	TC009589	KAK9758826.1	77%	366	77%	1 (10), 1 (11), 1 (17), 1 (23)
Tubulin alpha 1‐like (alphaTUB)	TC004873	KAK9729223.1	81%	—	—	—
Regulatory particle triple‐A ATPase 3 (RPT3)	TC007999	KAK9731517.1	81%	—	—	—

*Note*: The table lists the selected target genes to be silenced in *Popillia japonica*, the accessions of corresponding homologues in *Tribolium castaneum* iBeetle database and those of *P. japonica* proteins from the genome assembly (BioProject: PRJNA860365). The predicted identity of full‐length *P. japonica* coding sequences (CDS, detailed in Supporting Information, Table S1) with *Apis mellifera* homologues is indicated here. The table also indicates the length of designed *P. japonica* double‐stranded RNAs (dsRNAs), their predicted identity with *A. mellifera* homologues and numbers of retrieved stretches with identical consecutive nucleotides (indicated in parenthesis) on alignments between *P. japonica* dsRNAs and *A. mellifera* homologues.

### 2.3 RNA extraction and retro‐transcription

Total RNA was then extracted from single insects using a Direct‐zol RNA Mini Prep Kit (Zymo Research, Irvine, CA, USA) following the manufacturer's protocol with slight modifications indicated in Supporting Information Method S1. The optional DNAse step was also included, and finally the samples were eluted in DNAse/RNAse‐free sterile water (50 μL). Concentration, purity, and quality of RNA extractions were estimated by using a Nanodrop spectrophotometer 2000 (ThermoFisher Scientific, Waltham, MA, USA) and loading an aliquot of each sample (about 400 ng of total RNA) on agarose gel stained with ethidium bromide.

The high‐capacity cDNA Reverse Transcription Kit (Thermo Fisher Scientific) was used to retrotranscribe total RNA (30 ng) with random hexamers, according to the manufacturer's protocol.

### 2.4 Design and synthesis of dsRNA molecules

To reduce possible non‐target effects on useful insect species, *P. japonica* dsRNAs were designed in the gene regions less conserved through evolution, according to alignment with corresponding homologues of *A. mellifera* (Table 2). Several primer pairs were designed to amplify the selected portions of *P. japonica* genes, as well as for gene expression analysis by quantitative real‐time polymerase chain reaction (qPCR; Table S2). Primers used to generate the dsRNA templates included the T7 promoter sequence at their 5′‐end and were used to amplify *P. japonica* complementary DNA (cDNA). The PCR fragments were cloned as detailed in Method S1. The plasmids were used to *in vitro* synthetize dsRNAs using the MEGAscript RNAi Kit (Thermo Fisher Scientific), according to the manufacturer's instructions. A control template corresponding to a fragment of the gene sequence of green fluorescent protein (GFP), surely absent in insect genomes, was PCR‐amplified from plasmid pJL24^32^ and transcribed with MEGAscript Kit in parallel with *P. japonica* constructs. Synthetized dsRNAs were quantified using a Nanodrop spectrophotometer 2000 (Thermo Fisher Scientific). Integrity, purity and the expected size were checked by loading a 1 μL aliquot of each dsRNA on agarose gel stained with ethidium bromide.

### 2.5 *Ex vivo* dsRNA degradation assays by midgut juice

The assay was run to evaluate the possible degradation of dsRNAs in *P. japonica* midgut and hindgut, before performing the subsequent RNAi experiments. A method previously described^33^ was slightly modified as indicated in Method S1. Midgut or hindgut juice was extracted with two consecutive procedures (extractions a and b) from pooled organs of six adult females. The juice was incubated for 30, 60 and 120 min at room temperature together with dsRNAs targeting GFP. Furthermore, each juice extract (obtained from extractions a and b) was divided into three tubes (one for each incubation time) and added with 20 μL of dsGFP diluted at 100 ng μL^−1^, in a final condition of 2 μg of dsRNAs per treatment. As a control, the same amount of dsRNAs was incubated for the same times in 20 μL of double‐sterile water. To stop the degradation reaction, 2 μL of sodium dodecyl sulfate (SDS, 1%, *w/v*) was added and the integrity of dsRNAs was estimated by running the samples on 1% agarose ethidium bromide‐stained gel.

### 2.6 Methods of dsRNA delivery

#### 2.6.1 Microinjection bioassays

Larvae kept at 4 °C were microinjected between two abdominal segments under a stereomicroscope using a fine glass needle connected to a Cell Tram Oil microinjector (Eppendorf, Hamburg, Germany). Insects were injected with 1 μL of dsRNAs diluted in filter sterilized Tris–EDTA buffer at the concentration of 1 μg μL^−1^ (1 μg per insect). Injected grubs were singly placed in individual cells of silicone muffin trays, filled with soil collected at the sampling site, fed with strips of organic carrots and daily checked for survival. Gene expression analysis was carried out on alive insects at 7 days post injection (dpi) for the screening experiment (spring collection, *N* = 125), whereas at 7, 14 and 21 dpi for the over‐time silencing experiment (late summer collection, *N* = 58).

For the microinjection, adults were collected from the maintenance cage in a 50 mL tube and stored in ice until the treatment. The microinjection was performed by first making a guide hole with an insulin needle between two abdominal segments under a stereomicroscope, and then injecting 5 μL of dsRNAs diluted at the concentration of 600 ng μL^−1^ (3 μg per insect) using a micropipette equipped with an extra‐fine tip. The injected insects were placed in couples (one male and one female) in 300 mL disposable transparent plastic cups with lids and allowed to feed on detached grapevine leaves (one leaf per cup). Wet cotton was also put in the cups to maintain humidity. Insects were daily checked for survival. After 2 days, the same insect couples were transferred to other cups containing soil and newly emerged oat stems. After 6 days, couples were transferred into new cups with fresh soil and newly emerged oat stems for an additional 6 days. Insects were daily checked for survival. All alive insects (*N* = 23) were removed at 14 dpi and stored at −80 °C for further analyses.

#### 2.6.2 Plant‐mediated feeding bioassays

The two methodologies tested for administering dsRNAs to adults were (i) leaf discs and (ii) mass feeding. Before both feeding trials, adults were collected from the maintenance cage in a 50 mL tube and starved at room temperature for 2 h. In the meantime, round foliar discs (ten discs for each treatment) were isolated from grapevine leaves by pressing on the upper page with a 1.5 mL tube, avoiding the main veins. A drop of food glue was spotted on each leaf disc and then approximatelu 10 μL of dsRNA solution was pipetted in the glue drop to obtain 10 μg per disc. One hour later, leaf discs were dried and ready to be used for feeding assays. Starved adults were singly caged in disposable cups and allowed to feed on a treated leaf disc for 24 h in a climatic chamber.

For the mass feeding trial,1 mL of diluted dsRNAs was added to a grapevine leaf covered by food glue to reach 70 μg per leaf and was allowed to dry. Starved female adults (ten insects for each treatment) were allowed to feed on treated leaves in disposable cups for 24 h in a climatic chamber. Females were used to maximize the probability of leaf consumption because males tend to focus more on mating and may therefore feed less.^20^

In both cases (leaf discs and mass feeding), insects were then placed in couples (one treated female together with an untreated male) in disposable cups with soil and newly emerged oat stems as described earlier. Insects were daily checked for survival. All alive insects (*N* = 24 and *N* = 7 for leaf discs and mass feeding, respectively) were removed at 14 dpi and stored at −80 °C for further analyses.

### 2.7 Gene expression analysis

#### 2.7.1 Selection of reliable reference genes

Initially, the expression variability of the selected target genes (listed in Table 2) among different insect life stages was explored to possibly identify reliable housekeeping genes. Peritrophin‐A (homologue of *T. castaneum* TC011142) has been already used as RNAi target gene in a previous study^20^ and was included here as target transcript in the gene expression study. All the other genes were considered as possible references. For each analysed developmental stage (L1, L2, L3 and adult) two/four samples were analysed. The qPCR was run on cDNA synthesized as detailed earlier. The resulting cDNA was used as a template for qPCR as described in Method S1. The expression stability of candidate reference genes was calculated by CFXMaestro™ Software (Bio‐Rad, Hercules, CA, USA) with the Reference Gene Selection Tool.

#### 2.7.2 Expression analysis of transcripts targeted by dsRNAs

The qPCR was used to quantify the ability of the administered dsRNAs to silence target messenger RNAs (mRNAs). Thus, 3–15 biological replicates were analysed at each time point for each dsRNA‐treated group. For each sample, cDNA was synthesized as detailed earlier and used as a template for qPCR in the same conditions described in Method S1. The specificity of the PCR products was verified by melting curve analysis for all samples. No‐template controls were always included in each plate. Primers targeting signal recognition particle 54k, tubulin alpha 1‐like and V‐type proton ATPase subunit d 1‐like were used as reference genes to normalize the cDNA among samples. Normalized expression levels of each target gene for each sample were calculated by CFXMaestro™ Software (Bio‐Rad). The expression stability of reference genes was acceptable in the multiplate gene study.

### 2.8 Small RNA sequencing

Total RNAs extracted from pre‐wintering larvae sampled on the seventh day after the injection of dsRNAs targeting either the selected pest genes (regulatory particle non‐ATPase 6 (RPN) and shibire_dynamin‐like protein (SHI)) or the GFP were sent to Macrogen Inc. (Seoul, Republic of Korea) for sRNA library construction and sequencing. Four biological replicates were prepared for each condition, each containing a pool of three individuals. Briefly, libraries were prepared with SMARTer smRNA‐Seq Kit for Illumina (Takara Bio, San Jose, CA, USA) and sequenced by NovaSeq X (2 × 150). Raw reads were trimmed from adapters with Cutadapt, version 4.6,^34^ and filtered out according to (a) quality and length (minimum 19 nt; maximum 22 nt) and (b) mapping onto the *P. japonica* target coding sequences and onto the GFP coding sequence (control). Bowtie version 1.1.2 software^35^ with no mismatch allowed in the alignment was used to establish sRNA abundance profiles of the 12 sequenced samples. Following alignment, the resulting SAM files were converted to BAM format, sorted by position and indexed using SAMtools version 1.9^36^ and visualized with the Integrative Genomics Viewer (IGV).^37^ The sRNA counts were normalized for differences in sequencing depths to account for the technical differences across samples.

### 2.9 Data analysis

SigmaPlot version 15 (Systat Software, Inc., San Jose, CA, USA) was used for statistical analyses. Kaplan–Meier analysis was used to estimate the survival of *P. japonica* individuals subjected to different treatments, considering that insects could die (event of interest) or be censored (i.e., sampled at different time points for expression analyses). The log‐rank test was used to establish whether there was a statistically significant difference (*P* < 0.05) between the survival curves of each experimental condition. If significant differences occurred, Holm–Sidak method was used to quantitatively describe the difference between pairwise survival times. Survival plots were obtained using R version 4.1.3, R packages ‘survival’ and ‘survminer’. A *t* test or Mann–Whitney test, when the parametric analysis assumptions were not met, were used to compare levels of different transcripts measured as normalized expression in dsRNA‐ or dsGFP‐treated insects. The fold change reduction in gene expression for each target gene in comparison with the control dsGFP was determined as indicated in Method S1.

## 3 RESULTS

### 3.1 RNAi machinery genes are present in *P. japonica*

As a first step, to understand whether a functional RNAi machinery exists in *P. japonica*, the genome assembly (BioProject: PRJNA860365) was searched for genes involved in the RNAi pathway (Table 3). The presence of all major genes involved in the three main insect RNAi mechanisms (micro‐RNA (miRNA), small interfering RNA (siRNA) and piwi‐interacting RNA (piRNA) pathways) confirmed the presence of a potentially functional RNAi machinery in this species.

**Table 3:** RNA interference (RNAi) machinery genes

Gene names	*Popillia japonica* accessions	*Tribolium castaneum* accessions	Percentage identity	Query coverage
Ago1	KAK9736109.1	TC005857	99%	100%
Ago2	KAK9736108.1	TC011525	38%	88%
Ago3	KAK9686039.1	TC008511	47%	100%
Dicer1	KAK9718974.1	TC001750	66%	100%
Dicer2	KAK9694032.1	TC001108	48%	100%
Loquacious	KAK9692874.1	TC011666	65%	86%
piwi	KAK9686039.1	TC008711	33%	85%
R2D2	KAK9685513.1	TC008716	40%	98%

*Note*: Names and the accessions of corresponding homologues in *Tribolium castaneum* iBeetle database of the proteins involved in the insect RNAi pathways. Percentages of sequence identities and query coverage between *Popillia japonica* RNAi pathway proteins and the corresponding proteins from *Tribolium castaneum* are indicated.

### 3.2 Target genes and dsRNAs were selected to reduce off‐target effects

The nine candidate *P. japonica* selected genes (Table 2) were potentially involved in lethal phenotype, based on previous wide‐genome studies.^27^, ^29^, ^38^ To avoid off‐target effects according to the degree of gene conservation through evolution, *P. japonica* coding sequences were aligned with homologues of *A. mellifera*, used as representative of pollinators. Two candidate genes, namely tubulin alpha 1‐like and ATPase 3 showing over 80% identity with homologues of *A. mellifera*, were excluded. To further reduce non‐target effects, the portions of *P. japonica* genes chosen for dsRNA synthesis (ranging from 343 to 395 nt) were identified, according to alignments with corresponding homologues of *A. mellifera* (Table 2). Particular attention was paid to the number and the length of identical consecutive nucleotides longer than ten bases between *P. japonica* and *A. mellifera* gene fragments. Identical stretches of 19 nt, which may be critical and responsible for non‐target effects,^39^ were absent in all the selected portions, except for chosen fragments of signal recognition particle 54k and beta tubulin. Indeed, they displayed stretches of 21 and 23 identical consecutive nucleotides with *A. mellifera* homologues, respectively (Table 2).

### 3.3 The dsRNAs were not degraded in *P. japonica* midgut juice

**Figure 1:**
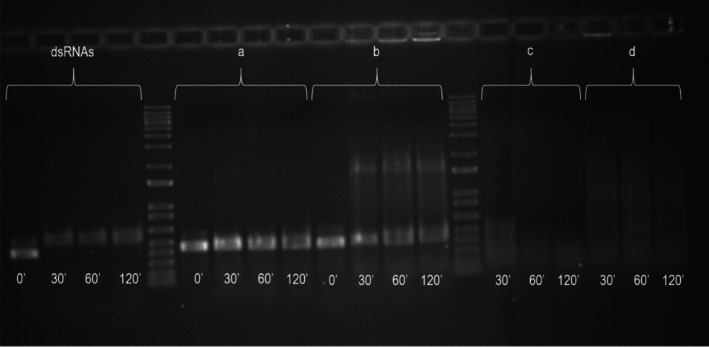
*Ex vivo* double‐stranded RNA (dsRNA) degradation assays by midgut juice. The dsRNA incubation in double‐sterile water or in midgut juice extracted from method a (uncrushed midguts (a) or hindguts (c)) or from method b (crushed midguts (b) or hindguts (d)) at different time intervals.

As a first step of RNAi experiments, an *ex vivo* dsRNA degradation assay by midgut juice of *P. japonica* adults was run to examine the possible persistence of dsRNA in the digestive canal. Figure 1 shows that no evident degradation of incubated dsRNAs occurred up to 2 h in both extraction conditions of midgut juice (method a, uncrushed tissues; method b, crushed tissues). However, dsRNAs were already completely degraded in 30 min when the intestinal extract was prepared including both midgut and hindgut (Fig. 1(c),(d)).

### 3.4 Three genes were selected as reliable housekeeping

All the nine selected *P. japonica* genes (Table 2) were assessed as possible references to be used as housekeeping in expression analysis. Supporting Information Fig. S1(a) shows the analysis of candidate reference genes, ranked from the most stable (in green on the left) to the least stable (in red on the right), to normalize the expression of peritrophin‐A among different insect life stages. Signal recognition particle 54k, tubulin alpha 1‐like and V‐type proton ATPase subunit d 1‐like were selected as the most reliable reference genes. The gene study to select reference genes also revealed that peritrophin‐A, which was already used as an RNAi target gene in a previous study,^20^ is over‐expressed in larval stages, but almost not expressed in adults (Fig. S1(b)).

### 3.5 Screening experiment: silencing of two genes increased mortality and all tested transcripts were reduced

**Figure 2:**
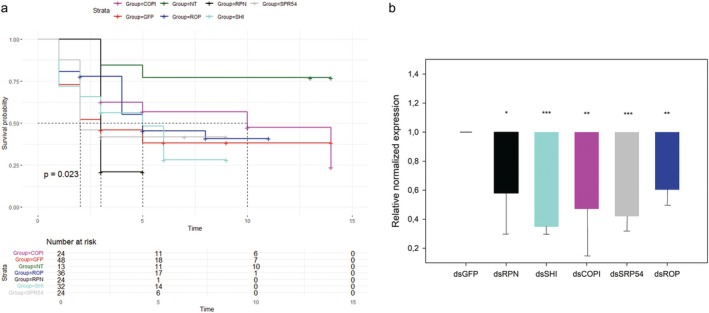
Screening experiment. Survival rates ((a) Kaplan–Meier log‐rank test) of *Popillia japonica* post‐wintering larvae either injected with double‐stranded RNAs (dsRNAs) targeting the selected genes (regulatory particle non‐ATPase 6 (RPN), shibire_dynamin‐like protein (SHI), coat protein β’ (COPI), signal recognition particle 54k (SRP54), ras opposite (ROP)) or the green fluorescent protein (GFP), or not treated (NT), up to 14 days. Numbers of treated insects are listed for each group. Mean relative normalized expression (b) of corresponding transcripts listed above in *P. japonica* post‐wintering larvae injected with dsRNAs targeting the selected genes in comparison with dsGFP‐treated insects. Error bars indicate standard error of the mean. A *t*‐test was used to compare expression level of corresponding transcripts in dsGFP‐insects with dsSHI‐ and dsSRP54‐treated samples, whereas Mann–Whitney rank sum test was used in the cases of dsRPN, dsCOPI and dsROP. Asterisks indicate significant differences in normalized expression levels between dsRNA‐injected insects against dsGFP‐treated insects; *, ** and *** indicate *P* < 0.05, *P* < 0.01, and *P* < 0.001, respectively.

Survival analysis revealed significant differences (log‐rank test, *P =* 0.023) among the mortality rates of treated post‐wintering larvae (Fig. 2(a) and Supporting Information, Dataset S1). There was a trend of reduced survival with dsRNAs targeting either the RPN or the SHI. For this reason, RPN and SHI were the targets selected for the subsequent trials.

A significant reduction of corresponding transcripts was measured for all target genes upon injection of specific dsRNAs in comparison with expression levels quantified in dsGFP‐treated insects (Fig. 2(b) and Dataset S2, *t‐*test used for SHI and signal recognition particle 54k, Mann–Whitney rank sum test used for RPN, coat protein β′ (COPI) and ras opposite (ROP)). In particular, the fold change reduction ranged from 2.2 (ROP) to 5.4 (COPI), confirming the effective gene silencing occurring after dsRNA treatment, for all the tested target genes.

### 3.6 RNAi silencing persisted up to 3 weeks after dsRNA‐treatment in over‐time experiment

**Figure 3:**
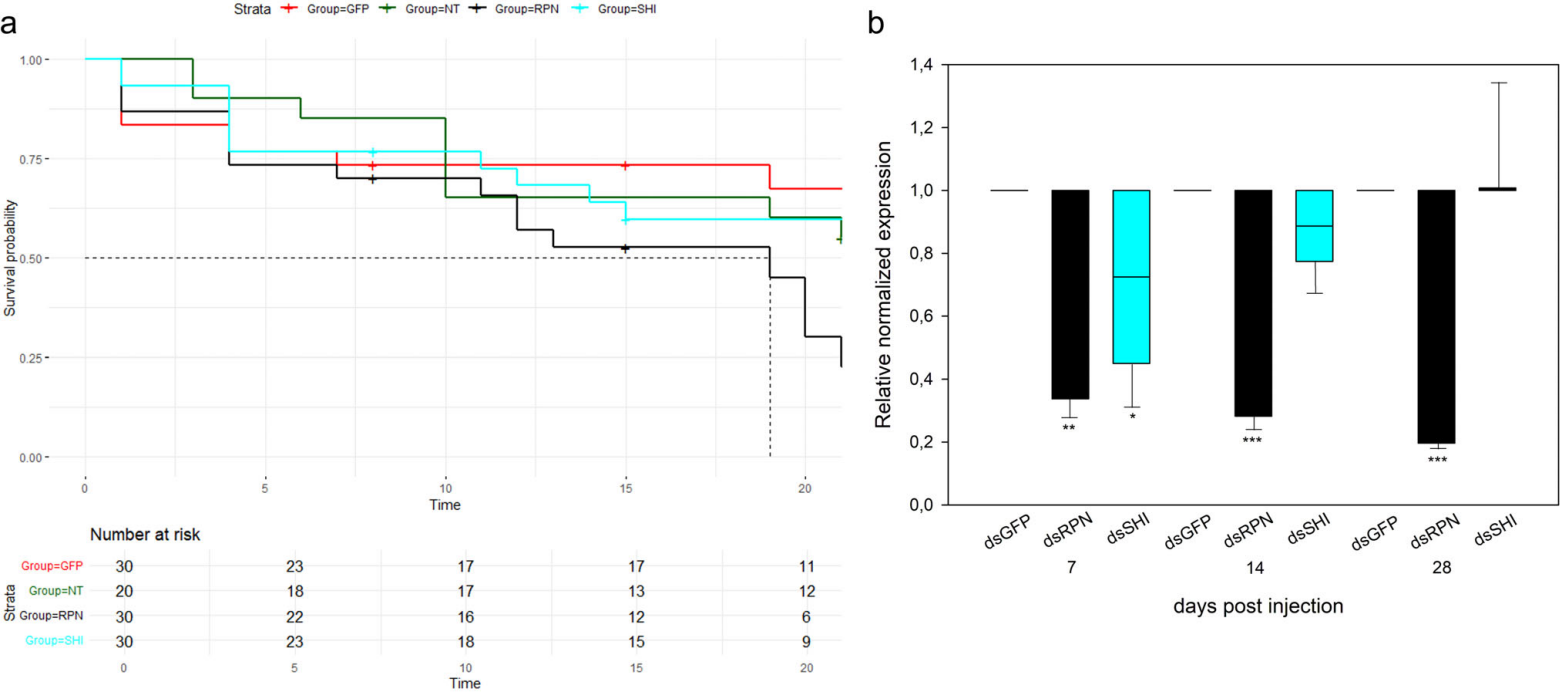
Over‐time silencing experiment. Survival rates ((a) Kaplan–Meier log‐rank test) of *Popillia japonica* pre‐wintering larvae either injected with double‐stranded RNAs (dsRNAs) targeting the selected genes (regulatory particle non‐ATPase 6 (RPN), shibire_dynamin‐like protein (SHI)) or the green fluorescent protein (GFP), or not treated (NT), up to 21 days. Numbers of treated insects are listed for each group. Mean relative normalized expression (b) of corresponding transcripts listed above in *P. japonica* pre‐wintering larvae injected with dsRNAs targeting the selected genes in comparison with dsGFP‐treated insects. Error bars indicate standard error of the mean. Mann–Whitney rank sum test was used to compare expression level measured at 7 days post injection (dpi), *t*‐test was used to compare expression data measured at 14 and 21 dpi. Asterisks indicate significant differences in normalized expression levels between dsRNA‐injected insects against dsGFP‐treated insects at the same sampling date; *, ** and *** indicate *P* < 0.05, *P* < 0.01, and *P* < 0.001, respectively.

According to the screening experiment, RPN and SHI were the best performing genes in inducing the highest mortality degree, once silenced. They were tested on pre‐wintering larvae to confirm the effect on survival rate of treated insects and assess the over‐time silencing efficiency. Survival analysis indicated that injection with dsRNAs targeting RPN induced a higher mortality rate than that observed in dsGFP‐treated insects (log‐rank test, *P =* 0.042) (Fig. 3(a) and Dataset S1).

A significant reduction of corresponding transcripts was measured for both target genes at 7 dpi in comparison with expression levels quantified in dsGFP‐treated insects (Fig. 3(b) and Dataset S2). In particular, fold change reductions of 3.3 (RPN, Mann–Whitney rank sum test, *P =* 0.008) and 2.9 (SHI, Mann–Whitney rank sum test, *P =* 0.032) were observed at the first sampling date. However, at 14 and 21 dpi, a significant transcript reduction was observed for RPN only (14 dpi: −3.7, *t*‐test, *P* ≤ 0.001; 21 dpi: −5.1, *t*‐test, *P =* 0.010), indicating a long‐lasting silencing effect, although not for all target genes.

### 3.7 Specific siRNAs were generated from injected dsRNAs

**Figure 4:**
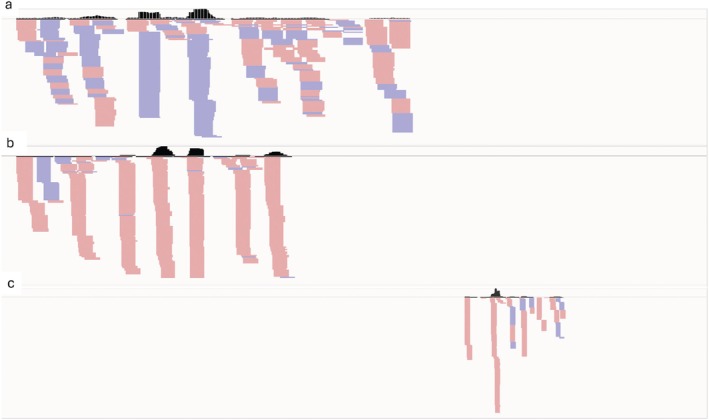
Small RNA (sRNA) profiles of dsRPN, dsSHI and dsGFP microinjected *Popillia japonica* pre‐wintering larvae. The sRNA mappings to the full‐length coding sequences of green fluorescent protein (GFP) ((a) 720 nt, replicate 3), regulatory particle non‐ATPase 6 (RPN) ((b) 942 nt, replicate 1), and shibire_dynamin‐like protein (SHI) ((c) 2694 nt, replicate 3) are reported. The black solid line corresponds to the position of the double‐stranded RNA (dsRNA) within each coding sequence. The abundance and peak distribution of sRNAs are indicated in the upper portion of each panel, while sense (red) or antisense (blue) mapping sRNAs are indicated in the lower portions.

For each injected dsRNA (dsRPN, dsSHI and dsGFP) four biological replicates were analysed for sRNA sequencing. In insects injected with dsRPN and dsSHI, the number of reads mapping to the sense strand of the cognate gene was at least 15 times higher than those mapping to the antisense, whereas in insects injected with dsGFP, the distribution of reads was the opposite, with a smaller difference in the number of reads mapping to the two strands (Table 4, Figs 4 and S2). The abundance of sRNA reads mapped to the three genes ranged from 0% to 1.9% of the total sRNA reads for each library, with only sRNA reads mapped to the RPN gene consistently above 0.5%, when insects were injected with dsRPN (Tables 4 and S3). Mapping of the sRNA reads to the three genes revealed an uneven distribution of the reads along the sequences (Fig. 4), with one or more peaks and no read mapping outside the region where the dsRNA was designed.

**Table 4:** Small RNA (sRNA) read counts

sRNA library names	Target gene	Read length (bp)	Number of mapped reads/strand orientation							
			Replicate 1		Replicate 2		Replicate 3		Replicate 4	
			Sense	Antisense	Sense	Antisense	Sense	Antisense	Sense	Antisense
dsRPN	RPN	24 622	19	244	24 256	352	15 704	0	16 977	44
		20	20 013	86	19 870	250	7661	22	14 376	50
		21	11 302	121	12 701	281	3393	5	8913	90
		22	15 714	131	17 446	224	4102	50	12 488	47
dsSHI	SHI	19	301	13	107	0	718	148	58	0
		20	350	12	245	0	1496	33	86	0
		21	468	0	104	0	1024	65	49	0
		22	381	17	307	0	962	28	108	0
dsGFP	GFP	19	33	57	57	75	319	479	60	291
		20	36	31	42	98	249	389	119	473
		21	48	126	81	209	329	722	149	867
		22	38	52	53	465	214	1104	143	1572

*Note*: Number of sRNA reads (length 19–22 nt) mapping to regulatory particle non‐ATPase 6 (RPN), shibire_dynamin‐like protein (SHI) or green fluorescent protein (GFP) gene in the 12 sRNA libraries analysed from the three groups of *Popillia japonica* pre‐wintering larvae injected with either dsRPN, dsSHI or dsGFP (four replicates each, 1–4), analysed at 7 days post injection. Numbers are normalized to account for differences in the number of total reads for each library and grouped by direction of the sense and antisense mapping to the coding sequence of the cognate gene.

### 3.8 Survival of *P. japonica* adults decreased after dsRNA‐treatment

**Figure 5:**
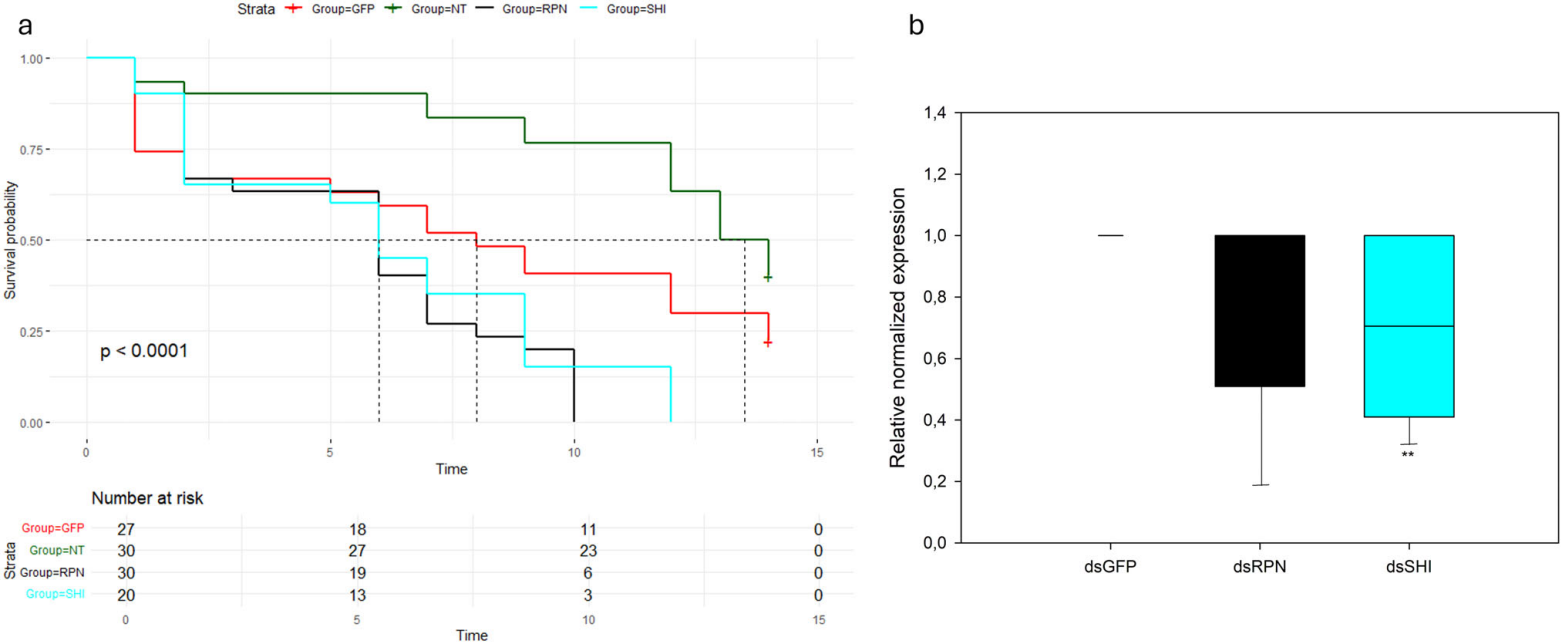
Injection of double‐stranded RNAs (dsRNAs) in adults. Survival rates ((a) Kaplan–Meier log‐rank test) of *Popillia japonica* adults either injected with dsRNAs targeting the selected genes (regulatory particle non‐ATPase 6 (RPN), shibire_dynamin‐like protein (SHI)) or the green fluorescent protein (GFP), or not treated (NT), up to 14 days. Numbers of treated insects are listed for each group. Mean relative normalized expression (b) of corresponding transcripts listed above in *P. japonica* adults injected with dsRNAs targeting the selected genes in comparison with dsGFP‐treated insects. Error bars indicate standard error of the mean. A *t*‐test was used to compare expression levels. Asterisks indicate significant differences in normalized expression levels between dsRNA‐injected insects against dsGFP‐treated insects; ** indicate *P* < 0.01.

Three delivery methods were attempted to administer dsRNAs to *P. japonica* adults: (i) abdominal injection, (ii) feeding on treated foliar discs, (iii) mass feeding on whole treated leaves. Survival analysis revealed significant differences (log‐rank test, *P =* <0.001) among the mortality rates of injected adults (Fig. 5(a) and Dataset S1). In particular, Holm–Sidak method indicated that injection with dsRNAs targeting RPN induced a higher mortality rate than that observed in dsGFP‐treated insects (*P =* 0.038). Consistently, a reduction of corresponding transcripts was observed in treated insects in comparison with dsGFP specimens (Fig. 5(b) and Dataset S2, *t*‐test).

No relevant effect was observed on survival rate (Fig. S3(a) and Dataset S1) as well as on transcript silencing (Fig. S3(b) and Dataset S2) in insects fed on dsRNA‐treated foliar discs, in line with the fact that most of the adults did not completely consume the entire leaf portion.

**Figure 6:**
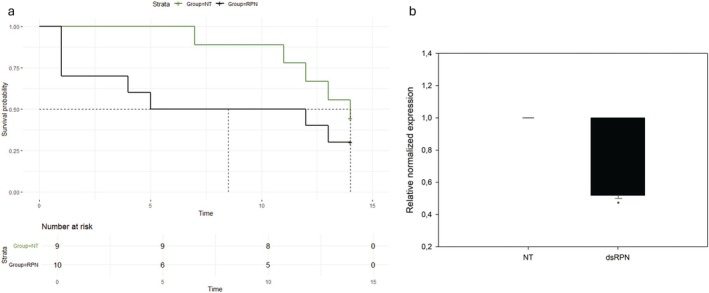
Mass feeding on double‐stranded RNAs (dsRNA)‐treated whole leaves. Survival rates ((a) Kaplan–Meier log‐rank test) of *Popillia japonica* adults mass fed on leaves either treated with dsRNAs targeting the selected gene (regulatory particle non‐ATPase 6 (RPN)) or not treated (NT), up to 14 days. Numbers of treated insects are listed for each group. Mean relative normalized expression (b) of RPN transcript in *P. japonica* adults injected with dsRNAs targeting the selected gene in comparison with untreated insects. Error bar indicates standard error of the mean. A *t‐*test was used to compare expression levels. Asterisks indicate significant differences in normalized expression levels; * indicate *P* < 0.05.

In a preliminary mass feeding assay on whole treated leaves, adults fed on dsRPN sprayed foliar tissue showed a median survival time of 8 days, whereas insects fed on untreated leaves averagely survived for 14 days (Fig. 6(a) and Dataset S1). Consistently, a significant reduction (*t*‐test, *P =* 0.032) of RPN transcript was measured in comparison with untreated insects (Fig. 6(b) and Dataset S2).

## 4 DISCUSSION

In the present study, several aspects of RNA interference in *P. japonica* were explored, with the long‐term aim of exploiting the best strategies to control the Japanese beetle. A list of the main core RNAi genes was retrieved from the recently re‐assembled Japanese beetle genome,^31^ showing that the RNAi mechanism occurs in this species. The most promising lethal genes for RNAi experiments were selected by injecting larvae with newly designed and synthesized dsRNAs. This procedure allowed us to monitor gene silencing over time and profile sRNAs generated by dsRNA treatment. The protocol optimized to treat larvae also made it possible to expand the experimental timeframe to study this univoltine species.^13^ The *ex vivo* degradation assay^33^ showed that dsRNAs remained intact in *P. japonica* midgut juice, indicating that feeding delivery was a strategy worth exploring. Therefore, a plant‐mediated assay was optimized to deliver dsRNAs to adults that were mass fed on treated leaves. This procedure showed results consistent with data obtained on larvae, confirming that the gene selection in grubs was suitable for the subsequent application to adults. Gene selection and dsRNA design to prevent possible target effects on *A. mellifera* demonstrated to be a practicable tool to implement sustainability into RNAi‐based approach, in a future provident perspective.

Nine *P. japonica* genes were initially selected to be screened as RNAi targets. This list was retrieved from unbiased genome‐wide screenings of valid RNAi targets in *T. castaneum*,^27^, ^29^ furthermore they were fine‐tuned by testing the most promising genes on other coleopteran species.^28^ To avoid undesirable side effects, two targets of *P. japonica* too similar to honeybee homologues (tubulin alpha 1‐like and ATPase3) were excluded from the initial list. Subsequently, dsRNAs targeting the remaining *P. japonica* seven genes were designed on the most divergent portions based. Effective gene silencing may be determined even only by a single stretch of 16 consecutive nucleotides in RNAi triggering molecules, either dsRNAs in Drosophila^39^ or siRNA in human kidney cells.^40^ Comparative analyses of different insect species showed that a minimum of at least three contiguous identical 21 nucleotides in homologous sequences is required to observe significant lethal or sublethal effects.^41^, ^42^ Here, consecutive stretches of more than ten identical nucleotides between dsRNAs targeting *P. japonica* genes and honeybee orthologues were highlighted and a cut‐off of 21 identical nucleotides was applied. Therefore, tubulin beta‐1 chain‐like, which showed one cluster of 23 bases identical to *A. mellifera* corresponding sequence, was excluded from successive trials. V‐type proton ATPase subunit d was also not included in the screening experiment, being one of the targets selected as housekeeping genes.

Silencing experiments on larvae revealed that all specific transcripts were significantly depleted, confirming that RNAi efficiently occurs in *P. japonica*. Silenced RPN and SHI increased insect mortality and thus both were selected for successive trials. The RPN is one of the subunits of the 26S proteosome multimeric complex, which is involved into the ubiquitin‐proteasome pathway and is responsible for the targeted degradation of damaged, misfolded, or mistranslated proteins in eukaryotic cells.^43^ This mechanism plays a major role in several cellular processes such as DNA damage repair, transcription, signal transduction, cell division and differentiation.^44^ It is noteworthy that the first sprayable dsRNA‐based insecticide against *L. decemlineata* targets a subunit of the 26S proteasome.^23^ In particular, the regulatory particles non‐ATPase of the 26S proteasome, including our RPN target, are responsible for recruiting polyubiquitinated proteins and directing them into the cylindrical component of the complex.^45^ Analyses of an RPN element insertion mutant revealed that this gene is essential for Drosophila development.^46^ Proteasome subunits were identified as one of the most efficient RNAi targets in genome‐wide screenings of *T. castaneum*.^27^, ^28^ Intriguingly, the 26S proteasome non‐ATPase regulatory subunit 6 specifically resulted to be the most RNAi effective and transferrable target among a selected list of over 40 genes tested in other insect species.^26^ As RPN is the most promising target selected in *P. japonica*, the potential off‐target effects were also examined for beneficial Coleoptera that are phylogenetically closer. The dsRPN of the Japanese beetle showed 67% identity with the homologue of *Coccinella septempunctata* (Coleoptera: Coccinellidae), lacking consecutive identical nucleotides of more than 15 bases. Shibire gene is the only Drosophila homologue to vertebrate dynamin, a mechanochemical enzyme involved in membrane‐remodelling events.^47^, ^48^, ^49^ Indeed, shibire gene product is a large GTPase mediating different endocytic pathways.^50^ In addition, it has pleiotropic effects on cell biology, such as actin reorganization,^51^ dynamic instability of microtubules,^52^ centrosome cohesion,^53^ and cytokinesis.^54^

Injection of dsRPN in pre‐wintering larvae (over‐time silencing experiment, Fig. 3) induced a significant increase in mortality, confirming RPN as promising RNAi target candidate. Moreover, a long‐lasting silencing effect was observed in injected larvae, as RPN transcript was still significantly down‐regulated up to 3 weeks after treatment. However, a case‐by‐case evaluation should be done to assess the specific silencing duration, as SHI transcript level returned to physiological levels as early as 2 weeks, even using the same dsRNA dose and identical treatment conditions as dsRPN. This result could be due to the abundance and turn‐over rate of a specific transcript as well as to any eventual positive/negative feedback mechanisms regulating transcription level.^26^ Interestingly, sRNAs mapping on RPN gene in dsRPN‐treated insects were averagely over 20 times more than what was observed in larvae injected with dsSHI (Tables 4 and S3), indicating that, regardless of the initial abundance of target transcript, dsRPN is able to trigger a stronger RNAi signal than dsSHI. However, maximum efficacy requires the rational design of dsRNA sequences, eventually supported by the recently developed *in silico* pipeline to enhance the efficacy and safety of RNAi‐based pest control strategies.^27^

The RPN was found to be a good RNAi candidate also in experiments with *P. japonica* adults, as its silencing increased mortality and reduced the transcript both after microinjection and mass‐feeding. Carroll *et al*. demonstrated that *P. japonica* adults, fed on artificial medium added with branched amphiphilic peptide capsules and complexed with dsRNAs targeting peritrophin, showed a reduction of the level of corresponding transcript, even though without a significant increase of mortality.^20^ It is noteworthy that, in our housekeeping gene selection, peritrophin was found to be poorly expressed in adults. This could also explain the scarce effect recorded by Carroll *et al*. after feeding on dsRNAs targeting this gene.^20^

Additional hints on RNAi mechanism in *P. japonica* may be deduced by the analysis of the sRNA patterns obtained from injected larvae in comparison with GFP treated controls. Total sRNA reads mapping to the dsRPN/dsSHI/dsGFP sequences in analysed libraries showed length distributions consistent with the processing of dsRNA into siRNA by the RNAi pathway, as previously observed in other insects.^55^ Interestingly, sRNA reads mapped exclusively to the fragment of the silenced transcript covering only the dsRNA portion sequence. The absence of read coverage over the remaining parts of the coding sequences confirms that mechanisms of secondary siRNA synthesis like those observed in plants and nematodes^56^ are absent in *P. japonica*. Similar results have been observed in other insect species,^57^, ^58^, ^59^ due to the absence of a functional RNA‐dependent RNA polymerase (RdRP), which has been observed only in the arthropod deer tick *Ixodes scapularis*, displaying four RdRP genes.^60^ Even though systemic RNAi is observed in insects, the specific mechanisms and the kind of signal molecules (dsRNA, siRNA or DNA) are yet to be unravelled.^19^ Interestingly, a comparative study on many different insect species indicated the ability of *P. japonica* to process dsRNAs into siRNAs, detected by a radio‐labelled probe, after injection but not after feeding of single larvae on treated small carrot slices.^61^

The *ex vivo* degradation assay indicated feeding as a feasible way to deliver dsRNAs to *P. japonica* adults, as dsRNAs remained intact after incubation in midgut juice for up to 2 h. In line with our results, absence of dsRNA degradation has been also observed by Carroll *et al*. in a buffer mimicking *P. japonica* gut juice.^20^ However, a comparative study on many different insect orders indicated *P. japonica* as one of the species showing the lowest concentration of body fluids required to completely degrade dsRNAs.^61^ Body fluids used by Singh *et al*. included hemolymph and lumen contents, presumably consisting of both midgut and hindgut.^61^ Consistently, in our degradation assay, when the intestinal extract was prepared including both midgut and hindgut, dsRNA degradation was already complete after only 30 min (Fig. 1(c),(d)), indicating that the experimental conditions were effective. However, this result is not discouraging, as based on ultrastructure observation, Coleoptera hindgut epithelium is a duct through which waste products from the digestive process are eliminated rather than a region for absorption and synthesis.^62^ Beside insect endonucleases, naked dsRNA molecules are prone to degradation by several biotic and abiotic stresses when used as pesticides in open field. Therefore, improved formulations (based on liposomes, chitosan, inorganic nanoparticles or branched amphipathic peptide capsules) as well as additional strategies (combination with nuclease inhibitors or chemically modification of the RNA) should be also considered to increase dsRNA bioavailability in cells.^19^

## 5 CONCLUSION

This work delved into RNAi occurring in the priority plant pest *P. japonica*, laying the groundwork for future applications of strategies based on such mechanism to control the Japanese beetle. RNAi‐based strategies are a promising approach to control the Japanese beetle, as they are highly selective and might be easily integrated with other more conventional tools. Issues related to delivery of dsRNAs, very common in some other pests (i.e., sap‐sucking insects), as well as to variability in RNAi‐efficacy, a shortcoming in the case of Diptera and Lepidoptera which are poorly responsive to RNAi, should not rise in *P. japonica*. Indeed, the Japanese beetle is a highly voracious chewing pest, and it belongs to Coleoptera, generally recognized as the order in which RNAi occurs most effectively. In this respect, the first sprayable insecticide based on dsRNAs to be marketed was against a chewing coleopteran, the Colorado potato beetle *L. decemlineata*. This product is based on naked unformulated dsRNAs targeting a subunit of the 26S proteasome complex, proving that even issues related to production costs can be overcome. Here, we identified a different subunit of the same multimeric protein (RPN) as a first good promising RNAi target candidate to start optimizing a dsRNA‐based insecticide against the Japanese beetle, proactively awaiting the European Union (EU) approval of this kind of strategy. In the context of sustainable agriculture, it is also worth emphasizing that the portion of *P. japonica* RPN gene on which dsRNA was designed excludes potential undesirable effects not only on the tested pollinator, but also on phylogenetically closer beneficial beetles.

## CONFLICT OF INTEREST

The authors declare that they have no known competing financial interests or personal relationships that could have appeared to influence the work reported in this article.

## AUTHOR CONTRIBUTIONS

All authors conceived the ideas and designed the experiments. GL performed the experimental work, GL and LG analysed the data, wrote the main draft of the manuscript and prepared the figures. All authors critically reviewed the manuscript and gave final approval for submission.

## Supporting information


**Table S1.** Accession numbers of selected target genes. The table lists the selected target genes to be silenced in *Popillia japonica*, the accessions of corresponding homologues in *Tribolium castaneum* iBeetle database, together with accessions of coding sequences (CDS) and proteins in *P. japonica* genome assembly (BioProject: PRJNA860365).
**Table S2.** Primer list. List of primers used in this work.
**Table S3.** sRNA read counts. Number of small RNA reads (length 19–22 nt) mapping to regulatory particle non‐ATPase 6 (RPN), shibire_dynamin‐like protein (SHI) or green fluorescent protein (GFP) gene in the sRNA libraries analysed from the three groups of *Popillia japonica* pre‐wintering larvae injected with either dsRPN, dsSHI or dsGFP (four replicates each, 1–4), analysed at 7 days post injection. Numbers are normalized to account for differences in the number of total reads for each library.
**Figure S1.** Selection of reference genes. Stability analysis of possible reference genes (a) and normalized expression level of peritrophin‐A (b) in larval (L1, L2, L3) and adult stages (A).
**Figure S2.** Small RNA profiles of dsRPN, dsSHI and dsGFP microinjected *Popillia japonica* pre‐wintering larvae. sRNA mappings (black solid lines) to the full‐length coding sequences of green fluorescent protein_dsGFP (a, 720 nt, replicates 1, 2, 4), regulatory particle non‐ATPase 6‐dsRPN (b, 942 nt, replicates 2, 3, 4), and shibire_dynamin‐like protein‐dsSHI (c, 2694 nt, replicates 1, 2, 4) are reported. The black solid line corresponds to the position of the dsRNA within each coding sequence. The abundance and peak distribution of sRNAs are indicated in the upper portion of each panel, while sense (red) or antisense (blue) mapping sRNAs are indicated in the lower portions.
**Figure S3.** Feeding on dsRNA‐treated foliar discs. Survival rates (a, Kaplan–Meier log‐rank test) of *Popillia japonica* adults fed on leaf discs treated either with dsRNAs targeting the selected genes (regulatory particle non‐ATPase 6‐RPN, shibire_dynamin‐like protein‐SHI) or the green fluorescent protein (GFP), or not treated (NT), up to 14 days. Numbers of treated insects are listed for each group. Mean relative normalized expression (b) of corresponding transcripts listed above in *P. japonica* adults fed on leaf discs treated with dsRNAs targeting the selected genes in comparison with insects fed on dsGFP‐treated foliar portions. Error bars indicate standard error of the mean. No significant difference was observed in normalized expression levels between dsRNA‐treated insects against dsGFP‐insects.

**Method S1.** Supplementary methods.

**Dataset S1.** Survival data of *Popillia japonica*.

**Dataset S2.** Normalized expression values.

## Data Availability

The data that supports the findings of this study are available in the supplementary material of this article.
