# Supplementary material for: RNAi‐mediated gene silencing of a 26S proteasome subunit increases mortality of the Japanese beetle Popillia japonica
Source: Pest Manag Sci. 2025 Oct 6;82(1):1066–78. doi: 10.1002/ps.70265 (PMC12713707; doi:10.1002/ps.70265)
Supplement: Supplementary file 1 — Table S1. Accession numbers of selected target genes. The table lists the selected target genes to be silenced in Popillia japonica, the accessions of corresponding homologues in Tribolium castaneum iBeetle database, together with accessions of coding sequences (CDS) and proteins in P. japonica genome assembly (BioProject: PRJNA860365). Table S2. Primer list. List of primers used in this work. Table S3. sRNA read counts. Number of small RNA reads (length 19–22 nt) mapping to regulatory particle non‐ATPase 6 (RPN), shibire_dynamin‐like protein (SHI) or green fluorescent protein (GFP) gene in the sRNA libraries analysed from the three groups of Popillia japonica pre‐wintering larvae injected with either dsRPN, dsSHI or dsGFP (four replicates each, 1–4), analysed at 7 days post injection. Numbers are normalized to account for differences in the number of total reads for each library. Figure S1. Selection of reference genes. Stability analysis of possible reference genes (a) and normalized expression level of peritrophin‐A (b) in larval (L1, L2, L3) and adult stages (A). Figure S2. Small RNA profiles of dsRPN, dsSHI and dsGFP microinjected Popillia japonica pre‐wintering larvae. sRNA mappings (black solid lines) to the full‐length coding sequences of green fluorescent protein_dsGFP (a, 720 nt, replicates 1, 2, 4), regulatory particle non‐ATPase 6‐dsRPN (b, 942 nt, replicates 2, 3, 4), and shibire_dynamin‐like protein‐dsSHI (c, 2694 nt, replicates 1, 2, 4) are reported. The black solid line corresponds to the position of the dsRNA within each coding sequence. The abundance and peak distribution of sRNAs are indicated in the upper portion of each panel, while sense (red) or antisense (blue) mapping sRNAs are indicated in the lower portions. Figure S3. Feeding on dsRNA‐treated foliar discs. Survival rates (a, Kaplan–Meier log‐rank test) of Popillia japonica adults fed on leaf discs treated either with dsRNAs targeting the selected genes (regulatory particle non‐ATPase 6‐RP [file PS-82-1066-s001.docx]

**SUPPLEMENTARY TABLES**

**Table S1. Accession numbers of selected target genes.** The table lists the selected target genes to be silenced in *Popillia japonica*, the accessions of corresponding homologues in *Tribolium castaneum* iBeetle database, together with accessions of coding sequences (CDS) and proteins in *P. japonica* genome assembly (BioProject: PRJNA860365).

| **Gene name** | **Abbreviation** | ***T. castaneum* accession** | ***P. japonica* accession (CDS)** | ***P. japonica***  **accession (protein)** |
| --- | --- | --- | --- | --- |
| regulatory particle non-ATPase 6 | RPN | TC006375 | JASPKY010000674.1:57865-142179 | KAK9686934.1 |
| shibire_dynamin-like protein | SHI | TC011058 | JASPKY010000058.1:546342-568052 | KAK9744413.1 |
| coat protein (coatomer) β' | COPI | TC013867 | JASPKY010000104.1:1113220-1119292 | KAK9737135.1 |
| ras opposite | ROP | TC011120 | JASPKY010000296.1:48303-55238 | KAK9710256.1 |
| V-type proton ATPase subunit d 1-like | vATPaseD | TC013357 | JASPKY010000386.1:200919-201965 | KAK9746943.1 |
| signal recognition particle 54k | SRP54 | TC002574 | JASPKY010000280.1:650232-676342 | KAK9711424.1 |
| tubulin beta-1 chain-like | betaTUB | TC009589 | JASPKY010000108.1:256509-257939 | KAK9758826.1 |
| tubulin alpha 1-like | alphaTUB | TC004873 | JASPKY010000162.1:1016742-1019563 | KAK9729223.1 |
| regulatory particle triple-A ATPase 3 | RPT3 | TC007999 | JASPKY010000131.1:545249-553481 | KAK9731517.1 |

**Table S2. Primer list.** List of primers used in this work.

| **Application** | **Primer name** | **5′-3′ sequences** | **Target gene** | **Prod. Size** | **qPCR efficiency** | **R^2^** | **Melt peak** |
| --- | --- | --- | --- | --- | --- | --- | --- |
| dsRNA synthesis | PJCopiF695_T7^#^ | **TAATACGACTCACTATAG**ATACGCAATCAGGGAAGCCT | copi coatomer subunit beta | 420 nt | / | / | / |
|  | PJCopiR1078_T7^#^ | **TAATACGACTCACTATAG**CCAACCCATAAACCAGTGCG |  |  |  |  |  |
|  | PJvATPaseA_F266_T7^#^ | **TAATACGACTCACTATAG**GACCTGGTATCTTGGGCTCT | V-type proton ATPase subunit A | 393 nt | / | / | / |
|  | PJvATPaseA_R622_T7^#^ | **TAATACGACTCACTATAG**CAGGCCACACTTGCAACATA |  |  |  |  |  |
|  | PJvATPaseD_F577_T7^#^ | **TAATACGACTCACTATAG**CTATGCAAAGAGATCGGCGG | V-type proton ATPase subunit D | 403 nt | / | / | / |
|  | PJvATPaseD_R943_T7^#^ | **TAATACGACTCACTATAG**ACGAATAAAACACCCCGAAATG |  |  |  |  |  |
|  | PJbetaTUB_F623_T7^#^ | **TAATACGACTCACTATAG**ACGATATCTGTTTCCGCACA | tubulin beta-1 chain-like | 402 nt | / | / | / |
|  | PJbetaTUB_R988_T7^#^ | **TAATACGACTCACTATAG**TCTGTTCGTCCACCTCCTTC |  |  |  |  |  |
|  | PJsrp54_F109_T7^#^ | **TAATACGACTCACTATAG**TTTGATGAAATGGCCGGTGG | signal recognition particle 54k | 384 nt | / | / | / |
|  | PJsrp54_R456_T7^#^ | **TAATACGACTCACTATAG**TTCTACGCCATCTTGAGCAA |  |  |  |  |  |
|  | PJshi_F904_T7^#^ | **TAATACGACTCACTATAG**TGAAGGACGCAATGTTTATAAGG | shibire_dynamin-like protein | 431 nt | / | / | / |
|  | PJshi_R1298_T7^#^ | **TAATACGACTCACTATAG**CTTCGCGTTTGATGGCTTCT |  |  |  |  |  |
|  | PJrop_F735_T7^#^ | **TAATACGACTCACTATAG**AGCTGAAGATTGTATGAAGGCT | ras opposite | 379 nt | / | / | / |
|  | PJrop_R1077_T7^#^ | **TAATACGACTCACTATAG**TCGTGGGACTGTGTATGGTT |  |  |  |  |  |
|  | PJRpn_F24_T7^#^ | **TAATACGACTCACTATAG**TCAAGGTTTAGAAAAGACTCCCA | regulatory particle non-ATPase 6 | 424 nt | / | / | / |
|  | PJRpn_R411_T7^#^ | **TAATACGACTCACTATAG**TCTGTGCCCTAACGATACTGT |  |  |  |  |  |
|  | T7GFPf^1^ | **TAATACGACTCACTATAG**CTTTTCACTGGAGTTGTCCC | Green Fluorescent Protein | 403 nt | / | / | / |
|  | T7GFPr^1^ | **TAATACGACTCACTATAG**GTTTGTGTCCGAGAATGTTTC |  |  |  |  |  |
| qPCR  target genes | PJCopi_F502^#^ | GTGAACGTCTCCCAGTAGCC | copi coatomer subunit beta | 110 nt | 96.9% | 0.994 | 78.0°C |
|  | PJCopi_R611^#^ | TTCGCCATCTCCACAAACGA |  |  |  |  |  |
|  | PJvATPaseA_F1198^#^ | TCCGTCTCAATCGTAGGAGC | V-type proton ATPase subunit A | 116 nt | 97.7% | 1.000 | 81.0°C |
|  | PJvATPaseA_R1313^#^ | TGGGCGAGCTTCTTATCCAA |  |  |  |  |  |
|  | PJvATPaseD_F370^#^ | CCAAAATGTCATCCGCTCGG | V-type proton ATPase subunit D | 114 nt | 95.8% | 0.999 | 79.0°C |
|  | PJvATPaseD_R483^#^ | AAAGAACGGGGCTAGTGGTG |  |  |  |  |  |
|  | PJbetaTUB_F225^#^ | AGTACGGTCTGGCCCTTTTG | tubulin beta-1 chain-like | 162 nt | 90.4% | 0.999 | 79.5°C |
|  | PJbetaTUB_R386^#^ | CAATCGCATCCTTCGGCTTC |  |  |  |  |  |
|  | PJsrp54_F586^#^ | ATGGATGCTACGATTGGTCAAG | signal recognition particle 54k | 150 nt | 106% | 0.987 | 78.0°C |
|  | PJsrp54_R735^#^ | AATTGGACTGTTTGTGGCTGC |  |  |  |  |  |
|  | PJshi_F459^#^ | GTACCGATAAAATGGCGCGC | shibire_dynamin-like protein | 214 nt | 103.5% | 0.997 | 80.0°C |
|  | PJshi_R672^#^ | CCTCTCGAACCGGTCTTCAC |  |  |  |  |  |
|  | PJrop_F199^#^ | ATGGAACGAATGGCTGAGCA | ras opposite | 165 nt | 95.0% | 0.999 | 79.5°C |
|  | PJrop_R363^#^ | AGGCCCTTCTCCCATTGTTG |  |  |  |  |  |
|  | PJRpn_F391^#^ | ACAGTATCGTTAGGGCACAGAC | regulatory particle non-ATPase 6 | 187 nt | 96.2% | 0.977 | 75.5°C |
|  | PJRpn_R577^#^ | CCCTCACAGCCATGCAGTAA |  |  |  |  |  |
|  | PJalphaTUB_F623^#^ | GATTCGATGGTGCGCTCAAC | tubulin alpha 1-like | 210 nt | 93.0% | 0.997 | 80.0°C |
|  | PJalphaTUB_R832^#^ | TGTATTTGCCGTGACGTGGA |  |  |  |  |  |
|  | PJPeritrof_F^2^ | GCTGGTACCTACTTCAATCC | peritrophin-A | 300 nt | 99.4% | 1.000 | 79.5°C |
|  | PJPeritrof_R^2^ | CATACAACCTGCATCTTCGG |  |  |  |  |  |

T7 promoter sequence is boldfaced. ^#^Primers designed in this work. ^1^Abbà et al., 2019. ^2^Carroll et al., 2023.

**Table S3. sRNA read counts.** Number of small RNA reads (length 19-22 nt) mapping to regulatory particle non-ATPase 6 (RPN), shibire_dynamin-like protein (SHI) or green fluorescent protein (GFP) gene in the sRNA libraries analysed from the three groups of *Popillia japonica* pre-wintering larvae injected with either dsRPN, dsSHI or dsGFP (four replicates each, 1-4), analysed at 7 days post injection. Numbers are normalized to account for differences in the number of total reads for each library.

| **sRNA library names** | **target gene** | **read length (bp)** | **Replicate1** | | **Replicate2** | | **Replicate3** | | **Replicate4** | |
| --- | --- | --- | --- | --- | --- | --- | --- | --- | --- | --- |
|  |  |  | **sense** | **anti-sense** | **sense** | **anti-sense** | **sense** | **anti-sense** | **sense** | **anti-sense** |
| dsGFP | RPN | 19 | 5 | 0 | 0 | 0 | 0 | 0 | 3 | 0 |
|  |  | 20 | 0 | 0 | 0 | 0 | 14 | 0 | 16 | 0 |
|  |  | 21 | 0 | 0 | 0 | 0 | 0 | 0 | 1 | 0 |
|  |  | 22 | 0 | 0 | 0 | 0 | 0 | 0 | 3 | 0 |
|  | SHI | 19 | 0 | 0 | 0 | 0 | 0 | 0 | 4 | 0 |
|  |  | 20 | 11 | 0 | 0 | 0 | 15 | 0 | 5 | 0 |
|  |  | 21 | 0 | 0 | 0 | 0 | 0 | 0 | 0 | 0 |
|  |  | 22 | 3 | 0 | 8 | 0 | 0 | 0 | 0 | 0 |
| dsRPN | GFP | 19 | 0 | 0 | 0 | 0 | 0 | 0 | 0 | 0 |
|  |  | 20 | 0 | 0 | 0 | 0 | 0 | 0 | 0 | 0 |
|  |  | 21 | 0 | 0 | 0 | 0 | 0 | 0 | 0 | 0 |
|  |  | 22 | 0 | 0 | 0 | 0 | 0 | 0 | 0 | 0 |
|  | SHI | 19 | 18 | 0 | 0 | 0 | 1 | 0 | 0 | 0 |
|  |  | 20 | 0 | 0 | 5 | 0 | 26 | 0 | 22 | 0 |
|  |  | 21 | 2 | 0 | 2 | 0 | 0 | 0 | 5 | 0 |
|  |  | 22 | 0 | 0 | 13 | 0 | 0 | 0 | 13 | 0 |
| dsSHI | GFP | 19 | 0 | 0 | 0 | 0 | 0 | 0 | 0 | 0 |
|  |  | 20 | 0 | 0 | 0 | 0 | 0 | 0 | 0 | 0 |
|  |  | 21 | 0 | 0 | 0 | 0 | 0 | 0 | 0 | 0 |
|  |  | 22 | 0 | 0 | 0 | 0 | 0 | 0 | 0 | 0 |
|  | RPN | 19 | 0 | 0 | 5 | 0 | 0 | 0 | 20 | 0 |
|  |  | 20 | 0 | 0 | 7 | 0 | 0 | 0 | 0 | 0 |
|  |  | 21 | 0 | 0 | 0 | 0 | 0 | 0 | 0 | 0 |
|  |  | 22 | 0 | 0 | 0 | 0 | 0 | 0 | 11 | 0 |

**SUPPLEMENTARY FIGURES**


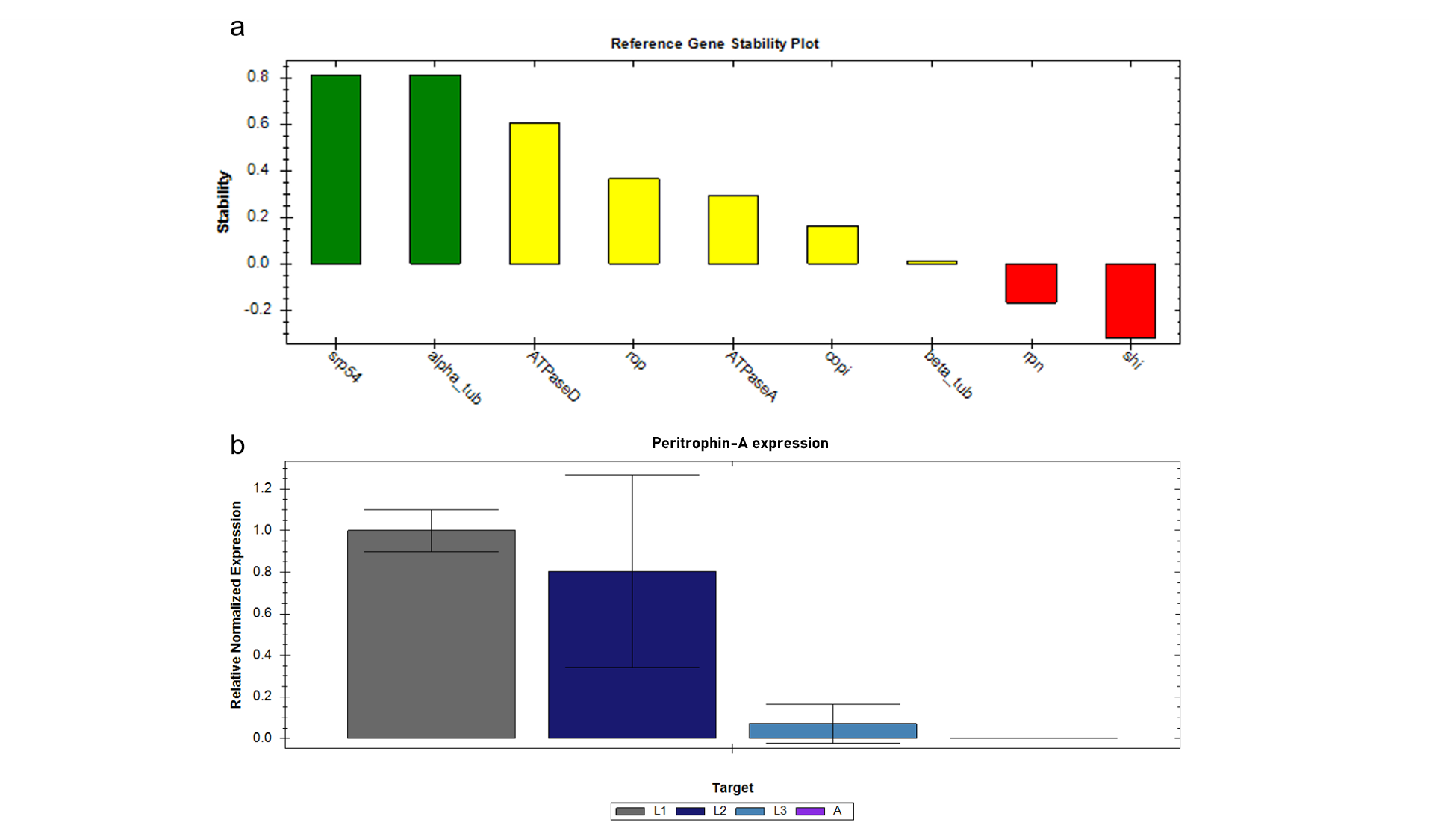


**Figure S1. Selection of reference genes.** Stability analysis of possible reference genes (a) and normalized expression level of peritrophin-A (b) in larval (L1, L2, L3) and adult stages (A).


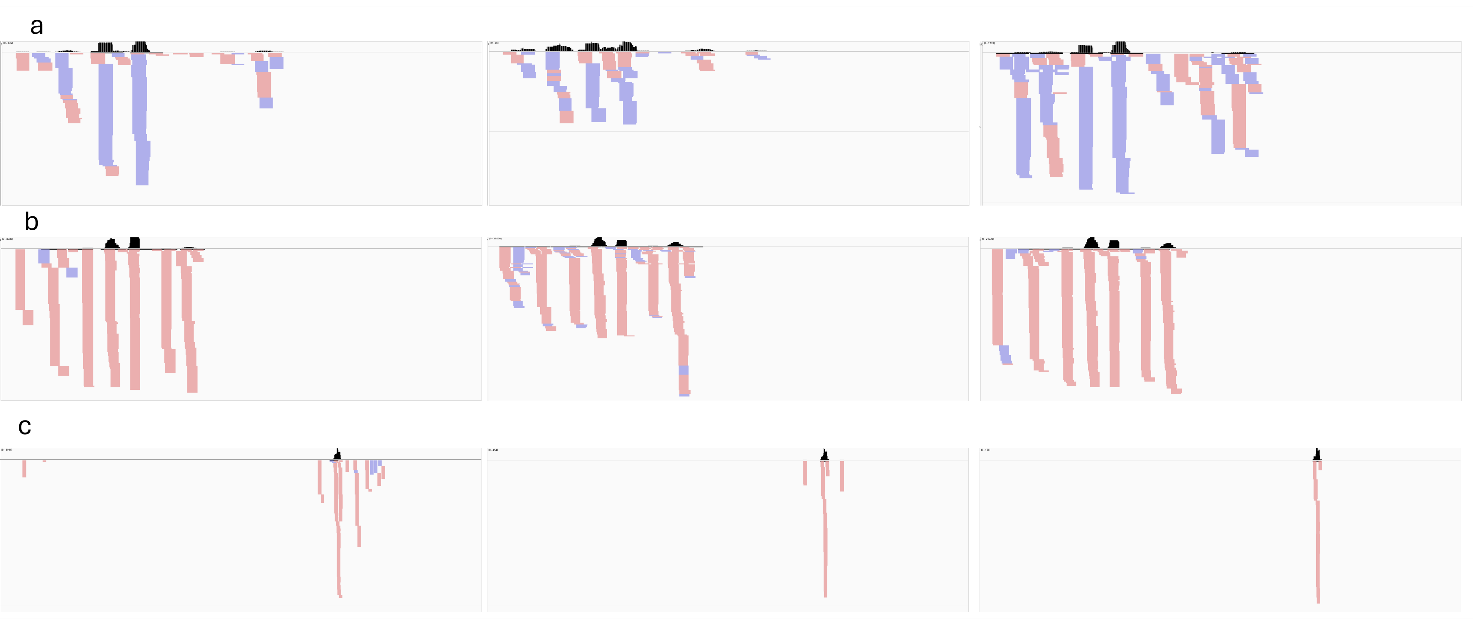


**Figure S2. Small RNA profiles of dsRPN, dsSHI and dsGFP microinjected *Popillia japonica* pre-wintering larvae.** sRNA mappings (black solid lines) to the full-length coding sequences of green fluorescent protein_dsGFP (a, 720 nt, replicates 1, 2, 4), regulatory particle non-ATPase 6-dsRPN (b, 942 nt, replicates 2, 3, 4), and shibire_dynamin-like protein-dsSHI (c, 2694 nt, replicates 1, 2, 4) are reported. The black solid line corresponds to the position of the dsRNA within each coding sequence. The abundance and peak distribution of sRNAs are indicated in the upper portion of each panel, while sense (red) or antisense (blue) mapping sRNAs are indicated in the lower portions.


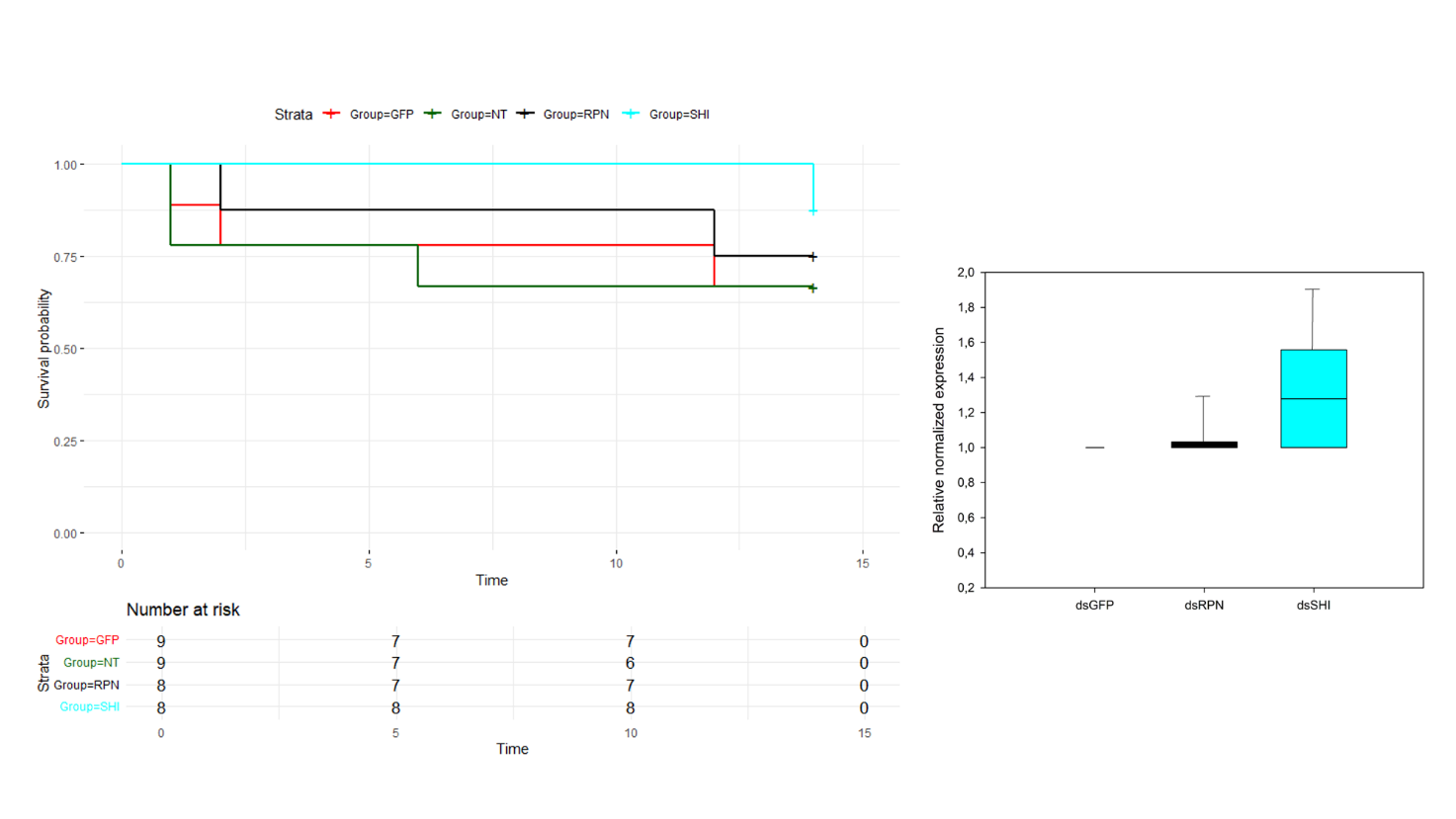


**Figure S3. Feeding on dsRNA-treated foliar discs.** Survival rates (a, Kaplan–Meier log-rank test) of *Popillia japonica* adults fed on leaf discs treated either with dsRNAs targeting the selected genes (regulatory particle non-ATPase 6-RPN, shibire_dynamin-like protein-SHI) or the green fluorescent protein (GFP), or not treated (NT), up to 14 days. Numbers of treated insects are listed for each group. Mean relative normalized expression (b) of corresponding transcripts listed above in *P. japonica* adults fed on leaf discs treated with dsRNAs targeting the selected genes in comparison with insects fed on dsGFP-treated foliar portions. Error bars indicate standard error of the mean. No significant difference was observed in normalized expression levels between dsRNA-treated insects against dsGFP-insects.
