# Supplementary material for: RNAi‐mediated gene silencing of a 26S proteasome subunit increases mortality of the Japanese beetle Popillia japonica
Source: Pest Manag Sci. 2025 Oct 6;82(1):1066–78. doi: 10.1002/ps.70265 (PMC12713707; doi:10.1002/ps.70265)
Supplement: Supplementary file 2 — Method S1. Supplementary methods. [file PS-82-1066-s004.docx]

**SUPPLEMENTARY METHODS**

**2.3 RNA extraction and retro-transcription**

For RNA extraction, each individual was rapidly transferred from -80°C storage in a screw-cap 2-ml tube together with about 40 zirconia/silica disposable beads (13 mm, BioSpec, nearly 20 beads on the bottom of the tube and 20 on top of the sample) and freeze-back in liquid nitrogen. Samples were crushed by bead beater (FastPrep-24, MP Biomedicals) in three homogenization cycles (1 min at maximum speed each), by storing samples in liquid nitrogen after each homogenization cycle. Total RNA was then extracted from single insects using a Direct-zol RNA Mini Prep Kit (Zymo Research) following the manufacturer’s protocol with slight modifications. Each crushed sample with zirconia/silica beads was added with 1.3 ml of TriFast II Nucleic Acids Isolation Reagent (Euroclone), strongly vortexed, incubated 5 min at room temperature and centrifuged for 1 min at max speed at 4°C. The supernatant (500 μl) was transferred in a clean 1.5 ml tube, added with 500 μl of fresh TriFast and centrifuged for 1 min at max speed at 4°C. The whole supernatant was transferred in a clean 2.2 ml tube, added with 1 ml of Ethanol 96%, vortexed and loaded on a Direct-zol RNA Mini Prep column, according to the protocol. The optional DNAse step was also included, and finally the samples were eluted in DNAse/RNAse-free sterile water (50 μl). Concentration, purity, and quality of RNA extractions were estimated by using a Nanodrop spectrophotometer 2000 (ThermoFisher Scientific) and loading an aliquot of each sample (about 400 ng of total RNA) on agarose gel stained with ethidium bromide.

To avoid successive inhibition in qPCR, the protocol for cDNA synthesis required some optimization steps. In the end, the High Capacity cDNA Reverse Transcription Kit (Thermo Fisher Scientific) was used to retrotranscribe total RNA (30 ng) with random hexamers, according to the manufacturer’s protocol.

**2.4 Design and synthesis of dsRNA molecules**

To reduce possible non-target effects on useful insect species, *P. japonica* dsRNAs were designed in the gene regions less conserved through evolution, according to alignment with corresponding homologues of *A. mellifera* (Table 2). Several primer pairs were designed to amplify of the selected portions of *P. japonica* genes, as well as for gene expression analysis by qPCR (Table S2). Primers used to generate the dsRNA templates included the T7 promoter sequence at their 5’-end and were used to amplify *P. japonica* cDNA. The PCR fragments were cloned in to pGEM-T Easy plasmid (Promega) by using *Escherichia coli* DH5α competent cells. Recombinant clones were purified with ZR Plasmid Miniprep™-Classic Kit (Zymo Research), Sanger-sequenced (BMR Genomics) to check integrity of T7 promoter sites and used as templates for the subsequent PCRs. One microgram (μg) of each column-purified PCR product (DNA Clean & Concentrator™-25, Zymo Research) was *in vitro* transcribed using the MEGAscript RNAi Kit (Thermo Fisher Scientific) according to the manufacturer’s instructions. A control template corresponding to a fragment of the gene sequence of green fluorescent protein (GFP), surely absent in insect genomes, was PCR-amplified from plasmid pJL24^32^ and transcribed with MEGAscript Kit in parallel with *P. japonica* constructs. Synthetized dsRNAs were quantified using a Nanodrop spectrophotometer 2000 (ThermoFisher Scientific). Integrity, purity and the expected size were checked by loading a 1 μl aliquot of each dsRNA on agarose gel stained with ethidium bromide.

**2.5 *Ex vivo* dsRNA degradation assays by midgut juice**

The assay was run to evaluate the possible degradation of dsRNAs in *P. japonica* midgut and hindgut, before performing the subsequent RNAi experiments. A method previously described^33^ was slightly modified as follows. Midgut or hindgut juice was extracted with two consecutive procedures (extractions a and b) from pooled organs of six adult females. Each *P. japonica* specimen was kept in ice for 5 min and then midguts or hindguts were dissected under a stereoscope in 1× phosphate-buffered saline (PBS). Organs dissected from the 6 insects were put altogether in a 1.5 ml tube and centrifuged at 1000 × *g* for 15 min at 4°C, to extract the juice. The supernatant was carefully collected (~10 μl) to avoid disturbing the pellet in a fresh 1.5 ml tube and rapidly used for the *ex vivo* dsRNA degradation assay (gut juice extraction a). Fresh PBS (10 μl) was added to the pellet and the mixture was further crushed with a micro-pestle and centrifuged again at 1000 × *g* for 15 min at 4°C, to extract a second portion of juice (gut juice extraction b). The supernatant was transferred (~10 μl) to a fresh 1.5 ml tube and used for the degradation assay. The juice was incubated for 30, 60 and 120 min at room temperature together with dsRNAs targeting GFP. In particular, each juice extract (obtained from extractions a and b) was divided into 3 tubes (one for each incubation time) and added with 20 μl of dsGFP diluted at 100ng/μl, in a final condition of 2 μg of dsRNAs per treatment. As a control, the same amount of dsRNAs was incubated for the same times in 20 μl of double-sterile water. To stop the degradation reaction, 2 μl of sodium dodecyl sulfate (SDS, 1%, w/v) was added and the integrity of dsRNAs was estimated by running the samples on 1% agarose ethidium bromide-stained gel.

**2.7 Gene expression analysis**

*2.7.1 Selection of reliable reference genes*

Initially, the expression variability of the selected target genes (listed in Table 2) among different insect life stages was explored to possibly identify reliable housekeeping genes. Peritrophin-A (homologue of *T. castaneum* TC011142) has been already used as RNAi target gene in a previous study^20^ and was included here as target transcript in the gene expression study. All the other genes were considered as possible references. For each analysed developmental stage (L1, L2, L3 and adult) two/four samples were analysed. Quantitative PCR (qPCR) was run on cDNA synthesized as detailed above. The resulting cDNA was used as a template for qPCR in a 10 μl volume mix, containing 5 μl iTaq Universal Sybr Green Supermix (Bio-Rad, Hercules, CA, USA) and 300 nM of each primer. All the primer pairs used for qPCR are listed in Table S2. Samples were run in duplicate in a CFX Connect Real-Time PCR Detection System (Bio-Rad). Cycling conditions were: 95 °C for 3 min, and 40 cycles at 95 °C for 15 s, and 60 °C for 30 s of the annealing/extension step. The specificity of the PCR products was verified by melting curve analysis for all samples. No-template controls were always included in each plate. The expression stability of candidate reference genes was calculated by CFXMaestro™ Software (Bio-Rad) with the Reference Gene Selection Tool.

*2.7.2 Expression analysis of transcripts targeted by dsRNAs*

Quantitative PCR was used to quantify the ability of the administered dsRNAs to knockdown target mRNAs. Three to fifteen biological replicates were analyzed at each time point for each dsRNA-treated group. For each sample, cDNA was synthesized as detailed above and used as a template for qPCR in the same conditions described above. The specificity of the PCR products was verified by melting curve analysis for all samples. No-template controls were always included in each plate. Primers targeting signal recognition particle 54k, tubulin alpha 1-like and V-type proton ATPase subunit d 1-like were used as reference genes to normalize the cDNA among samples. Normalized expression levels of each target gene for each sample were calculated by CFXMaestro™ Software (Bio-Rad). The expression stability of reference genes was acceptable in the multiplate gene study.

**2.9 Data analyses**

SigmaPlot version 15 (Systat Software, Inc., San Jose, CA, USA) was used for statistical analyses. Kaplan–Meier analysis was used to estimate the survival of *P. japonica* individuals subjected to different treatments, considering that insects could die (event of interest) or be censored (i.e., sampled at different time points for expression analyses). The log-rank test was used to establish whether there was a statistically significant difference (*P* < 0.05) between the survival curves of each experimental condition. If significant differences occurred, Holm-Sidak method was used to quantitatively describe the difference between pairwise survival times. T test or Mann–Whitney test, when the parametric analysis assumptions were not met, were used to compare levels of different transcripts measured as normalized expression in dsRNA- or dsGFP-treated insects. The fold change reduction in gene expression for each target gene in comparison with the control dsGFP was determined as follows. For each transcript, the mean expression of dsGFP-treated insects was set as one and the fold change values for each sample was calculated as ‒1 divided by the ratio between the normalized expression of that sample and the mean normalized expression of the GFP samples, according to following formula:

Fold change of treated sample X=‒1/(normalized expression of treated sample X/mean normalized expression dsGFP samples).
